# Do Attitudes, Mental Health Status, and Interpersonal Factors Predict COVID-19 Vaccine Hesitancy at the Early Phase of the Pandemic? A Longitudinal Study in Chinese College Students

**DOI:** 10.3389/fpsyg.2022.876116

**Published:** 2022-05-19

**Authors:** Zhipeng Wu, Xin Wang, Sha Zhang, Nani Ding, Guohua Zhang, Chengjia Zhao, Huihui Xu, Xinyi Lai, Xiaolian Tu, Xue Yang

**Affiliations:** ^1^School of Educational Science, Gannan Normal University, Ganzhou, China; ^2^Centre for Health Behaviours Research, JC School of Public Health and Primary Care, The Chinese University of Hong Kong, Hong Kong, Hong Kong SAR, China; ^3^School of Public Health and Management, Wenzhou Medical University, Wenzhou, China; ^4^The Affiliated Kangning Hospital, Wenzhou Medical University, Wenzhou, China; ^5^School of Mental Health, Wenzhou Medical University, Wenzhou, China; ^6^Renji College, Wenzhou Medical University, Wenzhou, China

**Keywords:** attitudes, mental health, social support, vaccine hesitancy, emerging adults, longitudinal study

## Abstract

**Purpose:**

The present study examined whether and how attitudes toward the COVID-19 vaccine (i.e., safety, efficacy, and price), mental health statuses (i.e., perceived stress and depression), and interpersonal factors (i.e., online social support, perceived social support) would predict COVID-19 vaccine hesitancy.

**Patients and methods:**

The two-wave longitudinal surveys were conducted in December 2019 and 2020 in Chinese medical college students (*N* = 194). Well- validated measures were used, including the Perceived Stress Scale, the Center for Epidemiologic Studies Depression Scale, the Online Social Support Questionnaire, and the Perceived Social Support Scale. Perceived safety, efficacy, price of COVID-19 vaccine, vaccine hesitancy, and actual intake were also measured.

**Results:**

Only 2.1% of participants had been vaccinated against COVID-19 in the early stages of the pandemic; 13.4% intended to get vaccinated in the next 3 months, and 66% intended to have it in the next 12 months upon follow-up. Multiple regression analyses revealed that perceived stress (βm = −0.15, *p* < 0.05) and depression (βm = −0.15, *p* < 0.05) were risk factors for positive attitudes toward the COVID-19 vaccine; online social support (ORm = 1.41, *p* < 0.01) and positive attitudes toward the COVID-19 vaccine (ORm = 1.83, *p* < 0.01) were protective factors of intention to get vaccinated in future.

**Conclusion:**

Findings suggest that intervention efforts should be made to reduce negative attitudes toward the COVID-19 vaccine among people with poor mental health and enhance online social support to promote COVID-19 vaccination.

## Introduction

The emergence of severe acute respiratory syndrome coronavirus 2, which causes the disease COVID-19, has had a destructive influence on global health and economy (Padron-Regalado, 2020). As of 2 January 2022, approximately 289 million cases and more than 5.4 million deaths were reported worldwide (World Health Organization, 2022). Vaccination may be one of the most effective strategies to slow down the spread of the disease. A large amount of research has indicated that COVID-19 vaccines show an obvious advantage in averting severe symptoms, hospitalization, and deaths (Tian et al., 2022). The public availability of vaccination is of vital importance, as community immunity can be established by large-scale vaccination programs (Fine et al., 2011). A recent study has shown that vaccine coverage of 55 to 82% among the population is required to establish community immunity to COVID-19 (Schaffer DeRoo et al., 2020). However, vaccination programs are threatened by growing vaccine hesitancy among the population. Evidence has shown that vaccine hesitancy can lead to a significant number of unvaccinated/under-vaccinated people, disease outbreaks, co-morbidities, as well as untimely deaths (Olusanya et al., 2021). Similarly, a study conducted in Hong Kong found that 39.7% of 1,047 adults were unsure about being vaccinated, and 19.9% reported being very unlikely to get vaccinated; The study also found that the increasing trend of vaccine hesitancy was largely responsible for the reduction of COVID-19 vaccine uptake (Wang K. et al., 2021). Therefore, due to the relatively high rate of vaccination hesitancy and its consequences, understanding its contributory factors is in urgent need.

Attitude refers to an individual’s overall feeling that the behavior is favorable or unfavorable (Ajzen, 2006). The Theory of Planned Behavior (TPB) posits that attitude is a critical factor behind decision-making by evaluating what motivates and inhibits people from adopting health behaviors (Ajzen and Driver, 1991). Perceived safety, efficacy and reasonable price of vaccine are particular important factors that dictate the decision among adults not to vaccinate to combat human papillomavirus (HPV) (Santhanes et al., 2018) and influenza (Bish et al., 2011). Therefore, these factors may also be applicable to understanding COVID-19 vaccination intention. Indeed, it has been reported that positive attitudes toward the vaccine against COVID-19, such as high levels of perceived efficacy and benefits of the vaccine, had a positive association with COVID-19 vaccine acceptance (Detoc et al., 2020; Faasse and Newby, 2020; Graffigna et al., 2020; Neumann-Böhme et al., 2020; Wang et al., 2020; Wong et al., 2020; Guidry et al., 2021; Kateeb et al., 2021; Riad et al., 2021b), while pessimism about side effects, vaccine efficacy and safety have been reported to increase COVID-19 vaccine hesitancy (Machida et al., 2021; Mo et al., 2021a; Skjefte et al., 2021; Hatmal et al., 2022). However, all these studies are cross-sectional. Few longitudinal evidence was found. So, more longitudinal studies are still needed.

Mental health problems, such as perceived stress and depression, are prevalent during the COVID-19 pandemic (Aristovnik et al., 2020; Abu Kwaik et al., 2021), which may have negative impacts on attitudes toward COVID-19 vaccination or intention toward vaccination (Mazereel et al., 2021). People with a poor mental health status may hold more pessimistic attitudes toward the vaccine and are less willing to get vaccinated. This may be because they are less likely to have self-efficacy in relation to health behaviors, and more likely to experience hopelessness and distress during the pandemic and adopt avoidance coping strategies instead of actively protecting themselves from being infected (Mazereel et al., 2021). Very limited studies have examined the associations between mental health status and attitudes toward the vaccine or intention toward vaccination against COVID-19, and the existing studies have reported inconsistent results. For example, one study found no significant relationship between mental health history and vaccine acceptance among the general adult population in Ireland (*N* = 1,041) and in the United Kingdom (*N* = 2,025) (Murphy et al., 2021). Another study with 32,361 adults in the United Kingdom found that those without long-term mental health conditions tended to express more specific worries about unexpected effects of vaccines and to have a higher preference for natural immunity (Paul et al., 2021). Studies on vaccinations for other diseases have also reported conflicting findings. For example, in a survey in Australia on the intention of individuals with schizophrenia to adopt preventive measures during the 2009 H1N1 influenza pandemic, 74% of respondents reported that they would be moderately willing to be vaccinated (Maguire et al., 2019). A pre-COVID-19 study in the United States reported that 84% of patients being treated for severe mental illness believed that, in general, vaccinations are safe, effective, and important (Miles et al., 2019).

Few studies have investigated whether interpersonal factors, such as social support, affect COVID-19 vaccination attitudes and intention. It has been revealed that social support can foster feelings of self-efficacy for particular health behaviors advocated by that social group (Guan and So, 2016). This phenomenon may be even more common in a collectivist culture where the family is central to identity (Wang C. et al., 2021). Emerging evidence also suggests that social support has been a critical precursor of engagement in preventive behaviors during the COVID-19 pandemic (Jetten et al., 2020). For example, a study among Japanese adults showed that loneliness (which is an indicator of low social support) was inversely associated with the likelihood of wearing a face mask, social distancing in public and handwashing (Stickley et al., 2020).

Linking with social networks, such as one’s neighborhood, closely, also allows access to risk and prevention information (Finnegan et al., 1993), enabling individuals to assess their risk effectively and take protective actions (Jaspal and Lopes, 2020). During periods of social distancing, social support has come in the form of many different “COVID-safe” ways (Nerlich and Jaspal, 2021). Having sufficient social support during COVID-19-associated isolation can be psychologically beneficial and conducive to better health outcomes, including adopting preventive behaviors such as getting vaccinated. Conversely, insufficient social support can lead to loneliness, which may limit information acquisition, action, and self-efficacy (Cacioppo and Hawkley, 2009; Stickley et al., 2020).

To the best of our knowledge, few studies have investigated how mental health and interpersonal status affect COVID-19 vaccination intention. Furthermore, it is essential to carry out a longitudinal study to monitor mental health and interpersonal status at different stages of the ongoing COVID-19 pandemic. However, no such studies have used a longitudinal design to identify their causal relationships. The current two-wave longitudinal study investigated the intention to get a COVID-19 vaccination and the potential influencing factors, including attitudes toward the COVID-19 vaccine (e.g., safety, efficacy, price), mental health statuses (e.g., perceived stress and depression), and interpersonal factors (e.g., social support), in a sample of Chinese college students. We hypothesized that negative attitudes toward the COVID-19 vaccine, a poor mental health status, and a poor interpersonal status predict a weak intention to get the COVID-19 vaccination.

## Materials and Methods

### Participants and Data Collection

This longitudinal study was originally designed to investigate medical college students’ stress and mental/behavioral health over time during their college life. Participants were recruited at Wenzhou Medical University in Wenzhou City, Zhejiang Province, China. The university has about 19,100 students. A total of 219 undergraduate students majoring in Anesthesia, Forensic, Oral medicine, and Traditional Chinese Medicine were invited to participate in the survey in December 2019 and 2020, respectively. Therefore, the baseline was a pre-COVID-19 survey, while the follow-up was conducted when the epidemic had been under control in China and the government started to promote COVID-19 vaccination. The inclusion criteria of this study were as follows: (1) being a college student; and (2) willing to participate in the baseline and follow-up studies. The exclusion criteria were as follows: (1) non-Chinese speaker; and (2) having a cognitive impairment that impeded the ability to understand the survey questions. Of the 219 participants who completed the baseline, 198 completed the follow-up. In addition, we found that four respondents had taken the vaccine; we excluded their data in the analyses because the number of this subgroup was too small for data analyses. As a result, data from the 194 participants was reported in this study.

The surveys were conducted in classroom settings. A research assistant with 2-year experience in data collection assisted with data collection. The respondents were assured that the participation was voluntary, and refusals would have no negative consequences. Participants were fully informed that their responses would be kept confidential and only the researchers could access the data. Data was matched by Student ID. Researchers were not able to access students’ identifying information (e.g., names). All respondents were briefed about the purpose and background of the study and provided their informed consent to participate in this anonymous survey. The study procedures were carried out in accordance with the Declaration of Helsinki. Ethical approval was obtained from the affiliated university of the corresponding author.

### Measures

#### COVID-19 Vaccine Outcomes at Follow-Up

At follow-up, attitudes toward the COVID-19 vaccine were measured using three questions, as follows: “To what extent do you agree that the COVID-19 vaccine is effective?”, “To what extent do you agree that the COVID-19 vaccine is safe (e.g., without severe side effects)?”, and “To what extent do you agree that the price of the COVID-19 vaccine is reasonable?”. Participants responded to these questions using a Likert-type scale ranging from 1 (strongly disagree) to 5 (strongly agree). Higher sum scores indicated more positive attitudes toward the COVID-19 vaccine. The scale had good reliability in the current sample (Cronbach’s alpha = 0.81).

To measure COVID-19 vaccine hesitancy/acceptance, participants were asked two questions: “Imagine that a vaccine against COVID-19 was available for anyone who wanted it at follow-up. Whether you would be likely to take the vaccine in (1) the next 3 months and (2) in the next 12 months?” (Robertson et al., 2021). Response options to the two questions were “yes” (1) and “no” (0). A higher sum score indicated a higher tendency of vaccine acceptance, while a lower score suggested a higher tendency of vaccine hesitance.

#### Mental Health Factors at Baseline and Follow-Up

At both baseline and follow-up, the 14-item Chinese version of the Perceived Stress Scale (Cohen et al., 1983; Hou et al., 2017) was used to measure perceived stress. An example item is “Feeling upset because something unexpected has happened and losing control of important things in life.” Each item is scored on a scale from 0 (never) to 4 (very frequently), with higher scores indicating a greater intensity of perceived stress. The Cronbach’s alpha for the scale was 0.80 at baseline and 0.78 at follow-up. The test-retest reliability was acceptable (intraclass correlation coefficient [ICC] = 0.75).

Depression was assessed using the Chinese version of the 20-item Center for Epidemiologic Studies Depression Scale (Cheung and Bagley, 1998). Participants rated how often they had experienced symptoms of depression, such as restless sleep and feeling lonely, in the past 7 days on a 4-point scale that ranged from 0 (rarely or never) to 3 (almost all of the time). The total score ranged from 0 to 60, with higher scores indicating more severe depressive symptoms. As is typically recommended, participants with a Center for Epidemiologic Studies Depression Scale score ≥ 16 were classified as having probable depression; this cutoff score is significantly associated with clinical assessments of depression (Radloff, 1977; Amtmann et al., 2014) and can predict depression diagnosis (Björgvinsson et al., 2013). The Cronbach’s alpha in the current sample was 0.89 at baseline and 0.90 at follow-up. The test-retest reliability was good (ICC = 0.77).

#### Interpersonal Factors at Baseline and Follow-Up

At both baseline and follow-up, the 23-item Online Social Support Questionnaire (Liang and Wei, 2008) was used to measure the perceived online social support of college students. An example item is “When you are feeling down or upset, you can get emotional support from your online friends.” Each item is scored from 1 (strongly disagree) to 5 (strongly agree), with higher scores indicating more online social support. The questionnaire is suitable for assessing online social support in the Chinese cultural context (Jiang, 2014; Wei et al., 2016). The Cronbach’s alpha for the scale was 0.76 at baseline and 0.78 at follow-up. The test-retest reliability was good (ICC = 0.71).

The 12-item Perceived Social Support Scale (Zimet et al., 1988; Yan and Zheng, 2006) was used to measure perceived general social support. Each item is scored from 1 (very strongly disagree) to 7 (very strongly agree), with higher scores indicating more perceived online social support. This scale is suitable for assessing perceived social support in the Chinese cultural context (Miao et al., 2016; Huang et al., 2020). The Cronbach’s alpha for the scale was 0.71 at baseline and 0.77 at follow-up. The test-retest reliability was acceptable (ICC = 0.70).

### Data Analyses

The proportions of endorsement of the items of attitudes toward the COVID-19 vaccine (“agree” or “strongly agree”) and COVID-19 vaccine hesitancy/acceptance (“yes”) at follow-up were reported. Linear regression analyses were performed to identify the background variables, mental health status, and interpersonal factors at baseline that were significantly associated with attitudes toward the COVID-19 vaccine (i.e., the sum score of the three questions of vaccine attitudes) at follow-up; the significant background variables of the outcomes (if any), mental health status, and interpersonal factors at follow-up were adjusted for in the linear regression analyses. Similarly, logistic regression analyses were performed to identify whether these factors and attitudes toward the COVID-19 vaccine were significantly associated with vaccine acceptance (i.e., endorsing the vaccine intention in the next 3 and/or 12 months) at follow-up, respectively; Standardized coefficients (β)/Odds ratio (OR) and 95% confidence interval (95%CI) were reported. All the analyses were performed using SPSS 21.0. The level of statistical significance was set at *p* < 0.05.

### Sample Size Calculation

Our primary aim was to investigate the predictors of COVID-19 vaccine acceptance. In the current multiple linear regression analyses, the R square explained by the five predictors was 0.127; the effect size f^2^ was 0.145. A *post hoc* power analysis using G-Power (version 3.1) indicated that a sample of 194 students would provide 99% power to detect an effect size f^2^ = 0.145 (α = 0.05, *F*-test: multiple linear regression model).

## Results

Of the participants (mean of age = 19.4), 59.8% were female; 52.1% were from urban areas; 47.9% lived in a one-child family; and 43.8% majored in traditional Chinese medicine (Table 1).

**TABLE 1:** Background characteristics of the participants (*N* = 194).

Socio-demographic variables		n	%
Sex	Male	78	40.2
	Female	116	59.8
Family origin	Urban	101	52.1
	Rural	93	47.9
One-child family	One-child	93	47.9
	More than one child	101	52.1
Subject/Major	Anesthesia	28	14.4
	Forensic	28	14.4
	Oral medicine	53	27.3
	Traditional Chinese Medicine	85	43.8
**Mental/interpersonal variables**		**Mean**	**Standard deviation**
Perceived stress	Baseline	36.38	6.66
	Follow-up	37.89	6.25
Depression	Baseline	19.20	6.48
	Follow-up	13.84	9.63
Perceived social support	Baseline	64.85	12.16
	Follow-up	64.91	11.38
Online social support	Baseline	78.36	15.38
	Follow-up	76.77	13.83

As shown in Table 2, 59.3% of participants considered that the COVID-19 vaccine is effective; most of the participants (60.3%) did not perceive the COVID-19 vaccine as safe; and 56.2% considered that the price of the COVID-19 vaccine is reasonable. Only 13.4% reported vaccine acceptance in the next 3 months, but 66% showed acceptance in the next 12 months.

**TABLE 2:** Proportion of positive attitudes toward the COVID-19 vaccine and vaccine hesitancy/acceptance at follow-up (*N* = 194).

Attitudes toward COVID-19 vaccine	n (%)
The COVID-19 vaccine is effective	115 (59.3)
The COVID-19 vaccine is safe	77 (39.7)
The price of the COVID-19 vaccine is reasonable	109 (56.2)
**COVID-19 vaccine hesitancy/acceptance**	
In the next 3 months	26 (13.4)
In the next 12 months	128 (66.0)
In the next 3 and/or 12 months	131 (67.5)

Background variables were not significantly associated with positive attitudes toward the COVID-19 vaccine or vaccine hesitancy/acceptance in simple regression analyses (*p* > 0.05). Therefore, no background variable was adjusted for in the following regression analyses.

As shown in Tables 3, 4, simple regression analyses revealed that perceived stress (βu = −0.15, *p* < 0.05) and depression (βu = −0.18, *p* < 0.05) were negatively associated with positive attitudes toward the COVID-19 vaccine. Online social support (ORu = 1.56, *p* < 0.01) and positive attitudes toward the COVID-19 vaccine (ORu = 2.03, *p* < 0.001) were positively associated with vaccine acceptance.

**TABLE 3:** Predictors of positive attitudes toward COVID-19 vaccine and vaccine hesitancy/acceptance by multiple linear regression models (*N* = 194).

Predictors	Positive attitudes toward COVID-19 vaccine		Vaccine hesitancy/acceptance	
		
	βu (95%CI)	βm (95%CI)	βu (95%CI)	βm (95%CI)
Perceived stress	−0.15*[−0.24, −0.05]	−0.15*[−0.29, −0.05]	−0.02[−0.02, 0.01]	−0.034[−0.02, 0.02]
Depression	−0.18*[−0.32, −0.08]	−0.15*[−0.25, −0.08]	0.06[−0.01, 0.02]	0.08[−0.01, 0.03]
Perceived social support	0.08[−0.01, 0.04]	0.14[−0.01, 0.06]	0.01[−0.01, 0.01]	−0.04[−0.01, 0.01]
Online social support	0.02[−0.02, 0.03]	0.01[−0.02, 0.03]	0.19** [0.002, 0.01]	0.18** [0.002, 0.01]
Positive attitudes toward vaccine	–	–	0.24***[0.03, 0.11]	0.22**[0.02, 0.10]

βu, Standardized univariate linear regression coefficients; βm, Standardized multivariate linear regression coefficients; 95% CI, 95% confidence interval; *p < 0.05, **p < 0.01, ***p < 0.001.

**TABLE 4:** Predictors of vaccine hesitancy/acceptance by multiple logistic regression model (*N* = 194).

Predictors	Vaccine hesitancy/acceptance	
	ORu (95%CI)	ORm (95%CI)
Perceived stress	1.06[0.97, 1.18]	1.04[0.96, 1.13]
Depression	0.93[0.90, 1.04]	0.95[0.91, 1.00]
Perceived social support	1.02[0.98, 1.05]	1.01[0.97, 1.05]
Online social support	1.56** [1.06, 2.31]	1.41** [1.01, 2.10]
Positive attitudes toward vaccine	2.03*** [1.40, 2.95]	1.83** [1.28, 2.62]

ORu, Standardized univariate logistic regression coefficients; ORm, Standardized multivariate logistic regression coefficients; 95% CI, 95% confidence interval; **p < 0.01, ***p < 0.001.

Similarly, multiple regression analyses showed that perceived stress (βm = −0.15, *p* < 0.05) and depression (βm = −0.15, *p* < 0.05) were negatively associated with positive attitudes toward the COVID-19 vaccine (Table 3). Online social support (ORm = 1.41, *p* < 0.01) and attitudes toward the COVID-19 vaccine (ORm = 1.83, *p* < 0.01) were positively associated with vaccine acceptance (Tables 3, 4).

## Discussion

The present study was conducted at an early phase of the pandemic when the Chinese government had just started promoting COVID-19 vaccines among relatively high-risk groups, such as healthcare or other workers providing care for patients with COVID-19, airport officials or public servants, and COVID-19 vaccines were not commercially available for general residents. In the current study, 67.5% of students had intention to uptake the COVID-19 vaccine in either 3 or 12 months. The prevalence of vaccine acceptance was comparable with previous studies conducted among university students (Riad et al., 2021a,c). However, only 13.4% of students intended to get vaccinated in the next 3 months. A possible explanation for this may be linked to the follow-up survey being conducted at an early stage in promoting COVID-19 vaccines; the students preferred to spend more time getting more information (e.g., the safety) about the vaccine.

We found significant positive relationships between positive attitudes toward the COVID-19 vaccine (i.e., “perceived the vaccine was effective, safe and with reasonable price”) and vaccine acceptance. These findings corroborate those of recent studies performed in various populations and cultures that suggested perceived efficacy and safety of the vaccine were positively associated with the likelihood of vaccination (Kateeb et al., 2021; Machida et al., 2021; Riad et al., 2021b; Skjefte et al., 2021). Such perceptions may be largely influenced by the fact that evidence on the efficacy and safety of the COVID-19 vaccine were not sufficient at this phase. A recent study reported that some Chinese citizens had mentioned worries over safety and efficacy as reasons for refusing vaccination (Yang, 2021). Moreover, China’s leading disease control official indicated that existing vaccines in China facilitated a low level of protection on 11 April 2021 (Yang, 2021). Another study also showed that 88.1% of the healthcare workers reported at least one side effect following the COVID-19 vaccination (e.g., injection site pain, headache/fatigue and muscle pain) (Klugar et al., 2021; Riad et al., 2021d). Our findings highlight the importance of enhancing vaccine efficacy and safety, as well as promoting public education on vaccine efficacy and safety to reduce vaccine hesitancy and increase acceptance.

In addition, a reasonable price may also encourage people to get vaccinated. At the time when this survey was conducted, there were debates about the market price of getting vaccinated and the local government had not officially announced the price. From 09 January 2021, vaccination is free of charge for Chinese citizens, which may have reduced vaccine hesitancy for some citizens. However, according to a report conducted in Beijing, the capital of China, 167.3 million doses of the vaccine were administered across the country; this is still far from the goal of vaccinating 560 million people (about 40% of the population), by the end of June 2021 (Yang, 2021). Another study among Chinese college students found that 78.9 and 60.2% of the participants endorsed the intention to get free and self-paid COVID-19 vaccination, respectively (Mo et al., 2021a). This suggests that the price may only affect the vaccine hesitancy of a small group of people. This group could be those with low-socioeconomic status as they may be more cost-sensitive (Wang J. et al., 2021). Hence, it is still important to guarantee that such vaccines are widely affordable to all socioeconomic groups.

One of the most key contributions of the current study is that we found mental health status and interpersonal variables predicted attitudes toward the COVID-19 vaccine and vaccine hesitancy/acceptance. People with poor mental health statuses (more severe perceived stress and depressive symptoms) were more likely to have pessimistic attitudes and responses toward the COVID-19 vaccine. The results are consistent with previous studies, which showed that people with poor mental health reported reduced intentions to engage in health-related behaviors (e.g., intention to exercise regularly and intention of medication adherence) (Manning and Bettencourt, 2011; Prugger et al., 2017). According to stress theories (e.g., the stress-vulnerability theory, the stress-coping theory), stress can elicit negative cognitive responses, such as catastrophizing and perceived loss of control, and also negative behavioral responses, such as avoidance behaviors (Lazarus and Folkman, 1984; Brown et al., 1995). These stress responses may explain the associations between poor mental health statuses and the negative attitudes toward the COVID-19 vaccine.

Our findings may highlight the need to pay more attention to those with poor mental/emotional status when promoting COVID-19 vaccination and other preventive behaviors in the general population. Prevention programs should make an effort to reduce negative feelings in the generation population. However, our result is inconsistent with those of recent COVID-19 studies that have reported non-significant relationships between mental health history and vaccine acceptance (Murphy et al., 2021) and negative associations between long-term mental health conditions and concerns about unforeseen effects of the vaccines (Paul et al., 2021). These inconsistent findings may be due to the fact that these studies investigated different types of mental health status and used different measures (e.g., self-report versus clinical diagnosis), study designs (e.g., longitudinal versus cross-sectional study design), and populations. Future studies should also examine the role of potential moderators (e.g., emotional disorders versus cognitive disorders, current mental health problems versus mental health history, clinical populations versus the general populations, and different cultures) to better understand the relationship between mental health and COVID-19 vaccine attitudes.

We found that online social support, rather than general social support, was a significant facilitator of vaccine acceptance. Since the COVID-19 outbreak, many cities in China have been placed under mass quarantine. Although social distancing is an important measure to slow down the spread of COVID-19, it reduces physical contact among friends and families and limits the exchange of general social support. Research has found that many people who are isolated from in-person social interactions turn to social media platforms for substituting online social support (Cellini et al., 2020). Furthermore, our participants were college students, for whom the Internet is the major source of social support. The Internet is also their major source of information (Baker et al., 2021). Our finding indicates that providing supportive and timely information related to vaccines (informational support) *via* the Internet may enhance vaccine acceptance among college students. This result also supports the theoretical model of social media-enabled healthcare for chronic disease, which asserts that different functions of social media are able to foster social support, where user creation affordance allows informational support. Social learning facilitates experiential support and user interaction facilitates emotional support. Such support is linked to self-care, health-related self-management, and psychological health (Lin and Kishore, 2021). Our study may extend the application of this model by highlighting that online social support may also facilitate preventive behaviors (e.g., vaccination) among healthy people. Future studies should investigate how these different types of online social support (e.g., informational support, emotional support, and experiential support) differentially affect preventive behaviors, such as vaccination, among healthy people.

One major limitation of this study is that we did not include actual behavior as an outcome and excluded those already vaccinated since the rate of actual vaccination behavior was very low when we conducted the survey. The early phase of the pandemic is a precious period to study the personal and interpersonal factors of vaccine hesitancy and vaccination intention with a minimized level of political influence. Indeed, the rate of vaccination has increased dramatically since July 2021 when local governments issued different local preventive measures to promote vaccination in China. For example, the residents’ access to schools, offices, or hospitals is restricted until they are vaccinated. At present, more than 1.2 billion people in Mainland China have received two doses of COVID-19 vaccines, accounting for 76.3% of the whole population (China Joint Prevention and Control Mechanism of the State Council, 2022). Collectivism culture together with control measures implemented by the local governments may be the key determinants for the success in vaccination coverage (Melton and Sinclair, 2021; Mo et al., 2021b). However, the personal and interpersonal factors we investigated may also help to understand vaccine hesitancy for other infectious diseases and COVID-19 vaccination in other cultures. Future studies should validate the findings for other infectious diseases and in other cultures. In addition, we only tested vaccine attitudes and hesitancy at follow-up, and thus did not test their changes at different stages of COVID-19. We did not conduct the second follow-up because, since 2021, most of the residents have been vaccinated due to disease control policy of local governments, and thus the personal and interpersonal factors might become less significant to predict one’s vaccine outcomes. The second limitation is that the study used a convenience sample majoring in Medicine, and the sample size was relatively small. Understanding the vaccine attitudes and hesitancy and their factors among medical students were particularly important as they may be important information sources for general people, but the results may not be able to be generalized to other populations. Caution is therefore needed when generalizing these findings to those of other ages or education backgrounds (e.g., majors, education levels). Third, participants were self-selected, and self-reported measures were used, which might induce report bias. Finally, this study used non-diagnostic measures to assess mental health status and a non-clinical sample. Future studies may validate our findings using clinical tools and patient samples.

## Conclusion

The present study found that most of the participants had hesitancy if they had to take the COVID-19 vaccine in the following 3 months. However, the rate decreased if they were to consider getting vaccinated in the next 12 months. Perceived stress and depression were significant risk predictors for positive attitudes toward the vaccine, and both positive attitudes toward the COVID-19 vaccine and online social support increased COVID-19 vaccine acceptance. These findings underscore the importance of promoting the efficacy and safety of the vaccine (both actual and perceived), reducing stress and depressive symptoms, and enhancing online social support in health behavior promotion among young people. The findings may facilitate vaccine promotion in a future pandemic.

## Data Availability Statement

The raw data supporting the conclusions of this article will be made available by the authors, without undue reservation.

## Ethics Statement

The studies involving human participants were reviewed and approved by the Ethics Committee of the Wenzhou Medical University. The patients/participants provided their written informed consent to participate in this study.

## Author Contributions

XY and ZW conceptualized the aims and hypotheses for the study. XY and XW were responsible for drafting the results and the manuscript. ZW and SZ assisted with manuscript editing. ND, GZ, CZ, HX, XL, and XT assisted with data collection. All authors contributed to the article and approved the submitted version.

## Conflict of Interest

The authors declare that the research was conducted in the absence of any commercial or financial relationships that could be construed as a potential conflict of interest.

## Publisher’s Note

All claims expressed in this article are solely those of the authors and do not necessarily represent those of their affiliated organizations, or those of the publisher, the editors and the reviewers. Any product that may be evaluated in this article, or claim that may be made by its manufacturer, is not guaranteed or endorsed by the publisher.
